# Ecogeographic variability and genetic diversity associated with seed albumins, globulins and prolamins patterns in *Vicia* taxa from Algeria

**DOI:** 10.1186/s40529-017-0177-7

**Published:** 2017-06-21

**Authors:** Sakina Bechkri, Imane Medoukali, Douadi Khelifi

**Affiliations:** Laboratoire de Génétique Biochimie et Biotechnologies Végétales, Faculté des sciences de la nature et de la vie, Université Frères MENTOURI, 25000 Constantine, Algeria

**Keywords:** *Vicia* L., Ecogeography, Electrophoresis, Albumins, Globulins, Prolamins

## Abstract

Genetic variability was studied in 78 populations of locally collected *Vicia* L. taxa for seed albumins, globulins and prolamins patterns by sodium dodecyl sulfate polyacrylamide gel electrophoresis (SDS-PAGE) along with an ecogeographic characterization of sites investigated. 131, 119 and 98 bands were respectively used for albumin, globulin and prolamin cluster analysis. Dendrograms based on the Jaccard index and the UPGMA method were generated and the degree of genetic diversity between and within taxa was evaluated. Five clusters were generated from albumins, six from globulins and four from prolamins patterns. The results reflect the great diversity of storage proteins and a high correlation was obtained between the three studied fractions. Several accessions present specific bands which could be used as a discriminatory marker both on intra and interspecific levels. No clear relationships were seen between the groups according to their geographical origin. Data obtained from ecogeographic investigation can be used for future collecting missions.

## Background

The genus *Vicia* belongs to the Legumes, family Leguminosae which is considered one of the largest families of flowering plants and represents tremendous morphological, ecological and genetic diversity. *Vicia* L. comprises about 210 species widely distributed along Europe, Asia and the American regions (Hanelt and Mettin 1989). In Algeria, there are 26 species belonging to three series (Quézel and Santa 1962). The genus *Vicia* has the capacity to fix atmospheric nitrogen (Nemecek et al. 2008). Vetch seeds contain more than 20% crude protein and relatively high amount of lysine, leucine, arginine, phenylalanine and tyrosine (Darre et al. 1998). Maxted (1993) pointed out that there had been 20 major classifications of the group since Linnaeus. Kupicha (1976) has subdivided the genus into two subgenera (*Vicilla, Vicia)* which have been further subdivided into 17 and 5 sections, respectively. The subgenus *Vicia* sensu Maxted (1993) contains 9 sections including sections Vicia, Hypechusa and Narbonensis. Section Cracca sensu Kupicha (1976) belongs to subgenus Vicilla. Morphological approach is rather difficult to estimate the all genetic diversity in the genus (Haider and El-Shanshoury 2000). Seed proteins are physiologically stable and easy to manipulate (Ladizinsky and Hymowitz 1979). Considerable insight has been drained as to their structure and synthesis during seed development and to their role as storage proteins (Higgins 1984). Electrophoretic analysis of seed storage proteins was used in testing genetic associations in *Vicia* at generic, specific and intraspecific levels, along with morphological characterization (Ladizinsky and Hymowitz 1979; Mirali et al. 2007; Hameed et al. 2009; Emre et al. 2010). The use of gel electrophoresis of seed protein in phylogeny is supported by the fact that mature seeds possess the same protein components unchanged with age or environmental stress, and thus provide valid evidence for genetic relatedness (Crawford 1990). Potokina et al. (2003) and Mirali et al. (2007) suggested that comparison of electrophoregrams of seed proteins is useful to assess relationships among *Vicia* taxa.

The objective of the present study was to investigate intra and interspecific variations in 11 taxa belonging to sections Vicia, Hypechusa, Narbonensis and Cracca by SDS-PAGE of seed albumins, globulins and prolamins to test the technique for vetches identification and to clarify the genetic diversity among *Vicia* taxa collected from different regions of the country along with an ecogeographic characterization of sites investigated as no studies have previously been reported on electrophoretic separation of the storage proteins of the given 11 *Vicia* taxa from Algeria.

## Methods

### Plant material and taxa identification

**Fig. 1:**
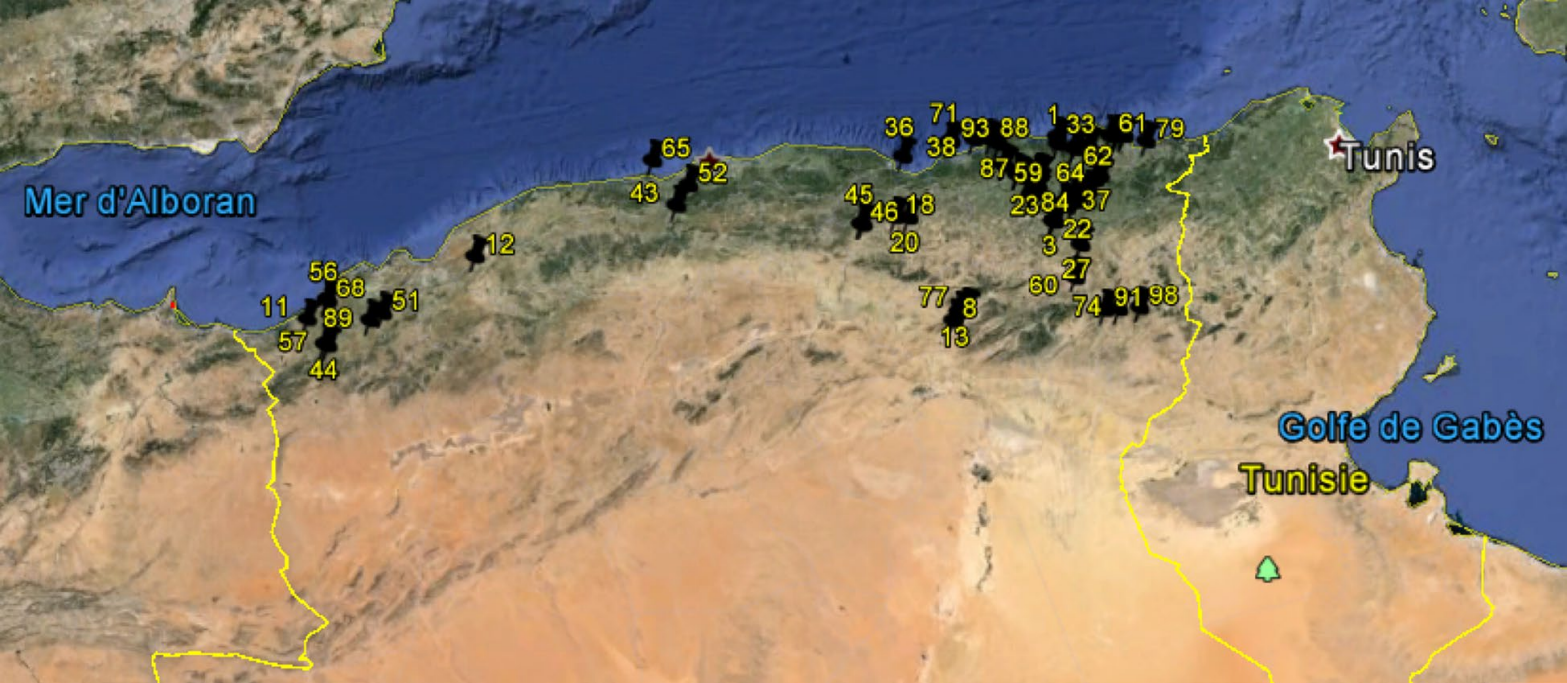
Geographical origin of the 78 Algerian populations studied

Object of the study were 78 accessions representing 4 taxa of Sect. Vicia, 2 taxa of Sect. Hypechusa, 1 taxon of Sect. Narbonensis and 4 taxa of Sect. Cracca. Pods were randomly collected from various bioclimatic conditions of Algeria (Fig. 1). The dry seeds were stored into separate sealed paper bags at room temperature until their utilization. Informations of the investigated accessions are given in Table 1. Taxonomic identification of accessions was verified by the morphology of plants grown from seeds in a greenhouse of the laboratory of genetics, biochemistry and plants biotechnologies of Faculty of Biology in Constantine University (eastern Algeria). Taxa identification was undertaken using the key of Quézel and Santa (1962).

**Table 1:** Location and taxonomic identification of accessions investigated

Species/subspecies		Code	Date of collection	Province/locality/origin	Latitude	Longitude	Altitude (m)
*V. sativa* subsp*. consobrina* (Pomel) Maire		5	28.5.14	Guelma	N36°26.187′	E007°17.772′	339
		14	1.6.14	Annaba El bouni	N36°49.777′	E007°38.290′	28
		36	23.5.14	Béjaia Affalou	N36°40.381′	E005°08.903′	1
		59	30.5.14	Jijel	N36°35.082′	E006°16.728′	141
		64	1.6.14	Skikda Azzaba	N36°43.532′	E007°04.706′	111
		86	13.6.14	Constantine Djbel El Ouehch	N36°23.690′	E006°39.011′	880
		65a	9.6.14	Tipaza El Beldj Chenoua mountain	N36°37.667′	E002°21.150′	345
		52	4.6.14	Blida National parc of Chréa	N36°24.538′	E002°45.519′	249
		53	30.5.14	Jijel	–	–	–
		85	1.6.14	Skikda Ain Charchar	N36°44.366′	E007°14.176′	52
		93	30.5.14	Jijel	N36°48.699′	E005°41.679′	25
*V. sativa* subsp*. obovata* Gaudin		6	22.5.14	Constantine Chaab ersas	N36°20.628′	E006°37.485′	563
		7	30.5.14	Mila Messaoud Boudjriou	N36°29.743′	E006°25.527′	325
		10	27.5.14	Constantine Didouche Mourad	N36°28.409′	E006°38.239′	468
		17	22.5.14	Constantine Chaab ersas	N36°20.628′	E006°37.485′	563
		20	3.6.14	Sétif Ain arnat	N36°07.394′	E005°12.172′	866
		22	2.6.14	Oum El Bouaghi Sigus	N36°04.485′	E006°48.867′	822
		28	30.5.14	Jijel	N36°35.094′	E006°16.732′	168
		51	6.6.14	Sidi Bel Abbes	N35°10.824′	W000°36.026′	490
		32	22.5.14	Constantine Chaab Ersas	N36°20.634′	E006°37.486′	562
		57	6.6.14	Tlemcen Ain fezza	N34°52.732′	W001°13.726′	867
		61	1.6.14	Annaba Berrahal	N36°49.826′	E007°29.000′	38
		68	6.6.14	Ain Temouchent	N35°16.464′	W001°13.836′	281
		72	28.5.14	Constantine Ain abid	N36°13.543′	E006°55.782′	847
		80	28.5.14	Constantine Ain abid	N36°13.543′	E006°55.782′	847
		70	28.5.15	Guelma	N36°14.816′	E007°03.045′	757
		83	26.5.14	Batna Ain Touta	N35°17.632′	E005°49.035′	683
*V. sativa* subsp*. angustifolia* (L.) Gaudin		19	18.5.14	Constantine Chaab Ersas	N36°20.634′	E006°37.486′	562
*V. sativa* subsp*. cordata* (Will) Batt.		8	26.5.14	Biskra El Kantra	N35°11.517′	E005°40.673′	467
		11	6.6.14	Tlemcen	N35°05.699′	W001°26.612′	90
		13	26.5.14	Biskra El Kantra Ain Skhoun	N35°16.087′	E005°44.174′	584
		15	1.6.14	Annaba	N36°49.980′	E007°34.092′	24
		33	29.5.14	Skikda El hadaik	N36°49.894′	E006°53.079′	26
		35	1.6.14	Annaba El bouni	N36°49.777′	E007°38.290′	28
		37	28.5.14	Guelma	N36°28.361′	E007°21.280′	223
		38	10.5.14	Jijel	N36°49.348′	E005°56.706′	14
		42	28.5.14	Constantine Ain Abid	N36°13.543′	E006°55.782′	847
		47	22.5.14	Constantine University	N36°20.387′	E006°37.177′	604
		71	30.5.14	Jijel	N36°47.625′	E005°39.746′	17
*V. lutea L.*							
*V. lutea* subsp*. vestita* (Boiss.) Rouy.		1	28.5.14	Skikda Ramdane Djamel	N36°45.977′	E006°53.432′	42
		4	22.5.14	Constantine University	N36°20.387′	E006°37.177′	604
		58	27.5.14	Constantine Didouche Mourad	N36°30.025′	E006°40.058′	448
V. *lutea* subsp. *eu*-*lutea* Maire		62	1.6.14	Skikda Azzaba	N36°43.531′	E007°04.708′	110
		63	1.6.14	Skikda Ain Cherchar	N36°44.366′	E007°14.176′	52
		79	1.6.14	El Tarf Ben M’hidi	N36°46.402′	E007°53.600′	11
		87	30.5.14	Jijel El Milia	N36°46.668′	E006°13.551′	28
		90	1st.6.14	Annaba	N36°49.980′	E007°34.092′	24
		88	30.5.14	Jijel El Ansar	N36°48.661′	E006°08.016′	30
		3	2.6.14	Oum El Bouaghi Sigus	N36°04.485′	E006°48.867′	822
		12	5.6.14	Relizane	N35°43.689′	E000°24.265′	105
		26	28.5.15	Guelma	N36°14.816′	E007°03.045′	757
		27	2.6.14	Oum El Bouaghi	N35°51.459′	E007°06.377′	887
		40	28.5.14	Guelma	N36°16.276′	E007°05.751′	711
		44	6.6.14	Tlemcen	N34°52.088′	W001°11.698′	843
*V. monantha* Retz							
*V. monantha* subsp*. calcarata* (Desf.) Maire		45	3.6.14	Bordj Bou Areridj	N36°04.070′	E004°41.899′	923
		49	28.5.14	Constantine Ain Abid	N36°13.543′	E006°55.782′	847
		60	2.6.14	Khenchla	N35°33.685′	E007°02.177′	860
		74	14.6.14	Tébessa	N35°15.936′	E007°30.306′	1078
		77	26.5.14	Batna Ain Touta	N35°17.632′	E005°49.035′	683
		78	20.5.14	Constantine Coudiat	N36°21.787′	E006°36.418′	633
		84	10.6.14	Constantine INATAA	N36°19.002′	E006°34.626′	586
		29	2.6.14	Oum El Bouaghi Sigus	N36°04.485′	E006°48.867′	822
		18	3.6.14	Bordj Bou Areridj Ain taghrout	N36°07.741′	E005°03.364′	934
		98	14.6.14	Tébessa Chria	N35°16.328′	E007°44.359′	1087
		102		Constantine University	N36°20.387′	E006°37.177′	604
		43	4.6.14	Médéa Oued Harbil	N36°13.633′	E002°37.643′	464
*V. monantha* ssp. *cinerea* (M.B.) Maire		46	3.6.14	Bordj Bou Areridj El Achir	N36°04.017′	E004°40.525′	944
		91	14.6.14	Khenchla	N35°15.704′	E007°20.957′	1222
*V. narbonensis* L.							
–		23	30.5.14	Constantine Hamma Bouziane (Chaabet El Medhbouh)	N36°26.391′	E006°33.282′	425
		30	30.5.14	Mila Messaoud Boudjriou	N36°29.748′	E006°25.530′	325
		34	27.4.14	Constantine Didouche Mourad	N36°29.216′	E006°38.731′	434
		41	28.5.14	Guelma	N36°16.276′	E007°05.751′	711
		55	27.5.14	Constantine Didouche Mourad	N36°30.023′	E006°40.051′	443
		66	28.5.14	Guelma	N36°19.930	E007°12.447′	698
		81	22.5.14	Constantine University	N36°20.387′	E006°37.177′	604
*V. tenuifolia* Roth.							
–		56	6.6.14	Ain Temouchent	N35°16.476′	W001°13.800′	276
		89	6.6.14	Sidi Bel Abbes Sidi Khaled	N35°06.59′	W000°44.238′	543
*V. leucantha* Biv.							
–		100	10.6.14	Constantine INATAA	N36°19.002′	E006°34.626′	586

### Protein sequential extraction based on solubility

The sequential extraction was undertaken according to Freitas et al. (2000) and Riberio et al. (2004) modified. Seeds were grinded to fine powder after seed coats were removed. 10 mg of the resulting flour was defatted with n-hexane (340 µl/10 mg) for 1/4 h with agitation, decanted and dried in stove (37 °C). Albumins were extracted with water (adjusted to pH 8.0) containing 10 mM CaCl_2_ and 10 mM MgCl_2_ (340 µl/10 mg) for ½ h. One mM phenylmethylsulphonyl fluoride (PMSF) was added to the extraction buffer. The content was centrifuged for 20 min. at 14,000 rpm (4 °C). The supernatant was recovered and the albumins were precipitated by acetone (561 g/l). Globulins were extracted by 0.1 M Tris–HCl buffer, pH 7.5–8, containing 10% (w/v) NaCl, 10 mM ethylenediaminetetraacetic acid (EDTA) and 10 mM ethyleneglycol bis (b-aminoethyl ether)-N, N, N0, N0-tetraacetic acid (EGTA) (340 µl/10 mg of flour) for ½ h. 1 mM phenylmethylsulphonyl fluoride (PMSF) was used to extraction buffer. The solution was centrifuged for 20 min. at 14,000 rpm (4 °C). Globulins were precipitated by acetone (561 g/l). Prolamins were extracted by 75% ethanol (50 µl/10 mg) for 20 min. with agitation at 4 °C. The prolamin-containing solution was centrifuged for 15 min. at 14,000 rpm (4 °C) and the prolamin were precipitated by acetone (500 µl).

### Electrophoresis

Non-reducing SDS-PAGE was undertaken according to Laemmli (1970). Bromophenol blue was added to the extraction buffer to follow proteins movement in the gel. 15, 8 and 70 μl of respectively albumins, globulins and prolamins supernatants were placed on biphasic polyacrylamide gels (12%). 10 μl of a protein molecular weight marker (BIO-RAD Precision Plus Protein Standards) containing ten proteins (10, 15, 20, 25, 37, 50, 75, 100, 150 and 250 kDa) was used as standard. Tris–glycine (pH 8.3) was used as electrode buffer. Runs were carried out at a voltage of 60 V and 500 mA overnight. Gels were stained by Coomassie Brilliant Blue R, then images were scanned using ImageScannerIII.

### Ecogeographic parameters of investigated sites

The five ecological factors of Mediterranean climate (annual rainfall, average of the maximum temperature of the hottest month, average of a minimum temperature of the coldest month, Emberger coefficient and altitude) were used to characterize sampling stations. A global positioning systems (GPS GARMIN eTrex^®^ model 30) was used to collect coordinates of sites investigated. Data recorded to ONM (National Office of Meteorology, Algeria) were used to characterize the climate of sites investigated (Table 2). Data recorded to CLIMATE-DATA.ORG (http://fr.climate-data.org/) were used for five stations (Mila, Ain Temouchent, Tipaza, El Tarf and Blida).

**Table 2:** Climatic characteristics of reference stations (2004–2014)

Reference station	Latitude	Longitude	Alt. (m)	P (mm)	m (°C)	M (°C)
Jijel (airport)	36°48N	05°53E	8	1066.1	6.8	31.5
Skikda	36°53N	06°54E	2	829	8.8	29
Annaba	36°50N	07°48E	3	684.4	6.7	31.5
Béjaia	36°43N	05°04E	2	833	7.4	30.7
Constantine	36°17N	06°37E	693	486.6	2.2	35.2
Mila^a^	36°27N	06°16E	437	742	4.4	31.5
Sétif (Ain Arnat)	36°10N	05°19E	1007	401.8	−2	34.8
Oum El Bouaghi	35°52N	07°07E	889	410.4	1.1	35
Tlemcen (zenata)	35°01N	01°28W	246	359.8	6.2	33.9
Ain Temouchent^a^	35°17N	01°08W	235	485	6.8	30.2
Biskra	34°48N	05°44E	82	143	6.9	41.3
Guelma	36°28N	07°28E	227	622.3	4.5	36.4
Sidi Bel Abbes	35°12N	00°37W	475	375.1	2.8	36
Tipaza El Beldj^a^	36°38N	02°21E	22	631	8.4	30.8
Blida^a^	36°27N	02°.44	1458	916	−0.9	28.5
Relizane	35°44N	00°32E	95	352.5	5.3	38.6
Bordj Bou Arerridj	36°04N	04°46E	928	392.9	1.7	36.4
Khenchla	35°28N	07°05E	983	520.8	1.8	34.9
Tébessa	35°25N	08°07E	821	382.6	1.7	35.6
Batna	35°45N	06°19E	822	346.8	0.1	36.4
Mila^a^	36°27N	06°16E	437	742	4.4	31.5
Ain Temouchent^a^	35°17N	01°08W	235	485	6.8	30.2
El Tarf Ben M’hidi^a^	36°46N	07°54E	6	707	7.1	31.2
Médéa	36°17N	02°44E	1030	780	3.5	32.5

*Alt* altitude, *P* annual rainfall, *M* and *m* are the average maximum temperature of the hottest month and the average of the minimum of the coldest month, respectively

^a^Data from “http://climate-data.org”

### Climatic data correction

Correction of precipitations and temperatures data based on extrapolations for different altitudinal points were undertaken (Table 3), according to the works of Seltzer (1946) as explained by Bechkri and Khelifi (2016).

**Table 3:** Corrected climatic data and Emberger quotient calculation of sampling sites of accessions studied

Station code	Alt. (m)	K	P (mm)	m (°C)	M (°C)	Q2	Bioclimate
5	339	1.0719	667.04	4.05	35.71	72.25	SH-temperate winter
14	28	1.0292	704.38	6.6	31.32	97.71	SH-mild winter
36	1	0.9990	832.16	7.40	30.70	122.48	SH-warm winter
59	141	1.0998	1172.49	6.26	30.56	165.49	LH-mild winter
64	111	1.1051	916.12	8.36	28.93	152.73	LH-warm winter
86	880	1.1537	561.39	1.45	33.89	59.35	HSA-cool winter
65a	345	1.4095	889.39	7.10	28.53	142.34	LH-warm winter
52	249	0.4720	432.35	3.93	36.96	44.90	MSA-temperate winter
93	25	1.0127	1079.63	6.73	31.38	150.23	LH-mild winter
6	563	1.1068	538.56	2.72	36.11	55.32	HSA-ool winter
7	325	0.9396	697.18	4.84	32.28	87.16	SH-mild winter
10	468	0.8150	396.57	3.1	36.77	40.39	MSA-temperate winter
17	563	1.1068	538.56	2.72	36.11	55.32	HSA-cool winter
20	866	0.8596	345.38	1.43	35.78	34.48	LSA-cool winter
22	822	0.9346	383.55	1.36	33.40	39.38	MSA-cool winter
28	168	1.1200	1194.03	6.16	30.38	169.09	LH-mild winter
32	562	1.1076	538.95	2.72	36.11	55.35	HSA-cool winter
51	490	1.0159	381.06	2.74	35.89	39.42	MSA-cool winter
57	867	1.6903	608.16	3.71	29.55	80.73	SH-temperate winter
61	38	1.0409	712.39	6.56	31.25	98.94	SH-cool winter
68	281	1.0379	503.38	6.61	29.87	74.22	SH-mild winter
72	782	1.0731	522.17	1.84	34.57	54.71	HSA-cool winter
80	782	1.0731	522.17	1.84	34.57	54.71	HSA-cool winter
70	757	1.3406	834.25	0.79	32.79	89.42	SH cold winter
83	683	0.8396	291.17	0.65	37.37	27.20	HA-cold winter
19	562	1.1076	538.95	2.72	36.11	55.35	HSA-cool winter
8	467	2.0769	296.99	5.36	38.60	30.64	HA-mild winter
11	90	0.8265	297.37	6.82	34.99	36.21	HA-mild winter
13	584	2.4041	343.78	4.89	37.78	35.84	HA-mild winter
15	24	1.0245	701.16	6.61	31.35	97.22	SH-mild winter
33	26	1.0231	848.14	8.70	29.53	139.67	SH-warm winter
35	28	1.0292	704.38	6.6	31.32	97.71	SH-mild winter
37	223	1.0025	611.62	4.51	36.52	65.53	SA-mild winter
38	14	1.0045	1070.9	6.77	31.45	148.82	LH-mild winter
42	847	1.1265	548.15	1.58	34.12	57.78	HSA-cool winter
47	604	0.9268	450.98	2.55	35.82	46.49	HSA-cool winter
71	17	1.0067	1108.4	6.76	31.43	154.13	LH-mild winter
85	52	1.0482	868.95	8.6	28.65	148.65	LH-warm winter
1	42	1.0386	860.99	8.64	28.72	147.07	LH-warm winter
4	604	0.9268	450.98	2.55	35.82	46.49	HSA-cool winter
58	448	0.7986	388.59	3.18	36.91	39.52	MSA-temperate winter
62	110	1.1042	701.16	8.36	28.24	120.97	SH-warm winter
63	52	1.0482	868.95	8.6	28.65	148.65	LH-warm winter
79	11	1.0056	710.93	7.08	31.16	101.27	SH-warm winter
87	28	1.0150	1082.09	6.72	31.36	150.63	LH-mild winter
90	24	1.0245	701.16	6.61	31.35	97.21	SH-mild winter
88	30	1.0165	1083.69	6.71	31.34	150.91	LH-mild winter
3	822	0.9346	383.56	1.36	35.46	38.58	MSA-cool winter
12	105	1.0113	356.48	5.26	38.53	36.75	LSA-mild winter
26	755	1.3393	833.44	2.38	32.80	93.97	SH-cool winter
27	887	0.9980	409.57	1.108	35.014	41.43	MSA-cool winter
40	711	1.3111	815.89	2.56	32.80	92.54	SH-cool winter
44	843	2.3274	837.39	3.81	29.72	110.85	LH-temperate winter
45	923	0.9949	390.89	1.72	36.43	38.63	MSA-cool winter
49	847	1.1265	548.15	1.58	34.12	57.78	HSA-cool winter
60	860	0.9055	471.58	2.29	35.76	48.33	HSA-cool winter
74	1078	1.2686	485.36	0.67	33.80	50.25	HSA-cold winter
77	683	0.8396	291.17	0.65	37.37	27.20	HA-cold winter
78	633	0.9506	462.56	2.44	35.62	47.82	HSA-cool winter
84	586	0.9120	466.85	2.62	35.94	48.06	HSA-cool winter
98	1087	1.2780	488.96	0.63	33.73	50.67	HSA-cold winter
102	604	0.9268	450.98	2.55	35.82	46.49	HSA-cool winter
43	464	0.7097	553.56	5.76	36.46	77.08	SH-mild winter
29	822	0.9346	383.56	1.36	35.46	38.58	MSA-cool winter
18	934	1.0061	395.29	1.67	36.35	39.10	MSA-cool winter
46	944	1.0162	399.26	1.63	36.28	39.52	MSA-cool winter
91	1222	1.1835	616.36	0.84	33.22	65.29	SH-cold winter
23	425	0.7796	379.35	2.62	35.94	39.05	MSA-cool winter
30	325	0.9396	697.18	4.84	32.28	87.15	SH-mild winter
34	434	0.7870	382.95	3.23	37.01	38.88	MSA-temperate winter
41	711	1.3111	815.89	2.56	33.12	91.58	SH-cool winter
55	443	0.7944	386.55	3.2	36.95	39.28	MSA-temperate winter
66	698	1.4	871.22	2.61	33.20	97.68	SH-cool winter
81	604	0.9268	450.98	2.55	35.82	46.49	HSA-cool winter
56	276	1.0338	501.38	6.63	29.91	73.87	SH-mild winter
89	543	1.0725	402.29	2.52	35.52	41.81	MSA-cool winter
100	586	0.912	466.85	2.62	35.94	48.06	HSA-cool winter

*K* correction factor, *Alt* altitude, *P* annual rainfall, *M* and *m* the average maximum temperature of the hottest month and the average of the minimum of the coldest month, respectively, *Q2* Emberger coefficient, *SH* subhumid, *LH* less humid, *HSA* higher semiarid, *HA* higher arid, *MSA* means semiarid, *LSA* less semiarid, *SA* semiarid

### Calculation of the bioclimatic coefficient of Emberger (1955) and definition of the bioclimate

The pluviothermic Emberger quotient (Q2) is determined by three major climate factors. Stewart’s formula (1969) was used in the present study. Details of calculations were reported by Bechkri and Khelifi (2016).

### Data analysis

The mobility and the frontal report of each band were calculated. The size marker standard curve was traced. The graphical equation and the coefficient of determination allowed the calculation of the molecular weight of each band. In this method, “absence” contributed equally to “presence” in the calculation of dissimilarity. Present bands were scored 1 and absent bands were scored 0. For each fraction, a binary matrix was constructed. A dendrogram was produced by the UPGMA based on Jaccard index (J) between protein patterns. Analyses were carried out using XLSTAT (Pearson edition, version 2014.5.03). For ecogeographic parameters, Euclidean distances (Romesburg 1990) were used in the estimation of the genetic resemblance. Matrix including the five ecological parameters of each accession was used to elaborate a dendrogram using UPGMA. Analyses were carried out with STATISTICA (version 6.1 program). The possible correlation between albumins, globulins and prolamins patterns, was evaluated by a Mantel test (Mantel 1967) based on Pearson’s correlation (XLSTAT Pearson edition, version 2014.5.03). The same test was used to test geographical matrix with seed albumins, globulins and prolamins matrices.

## Results

### Seed proteins variability

**Fig. 2:**
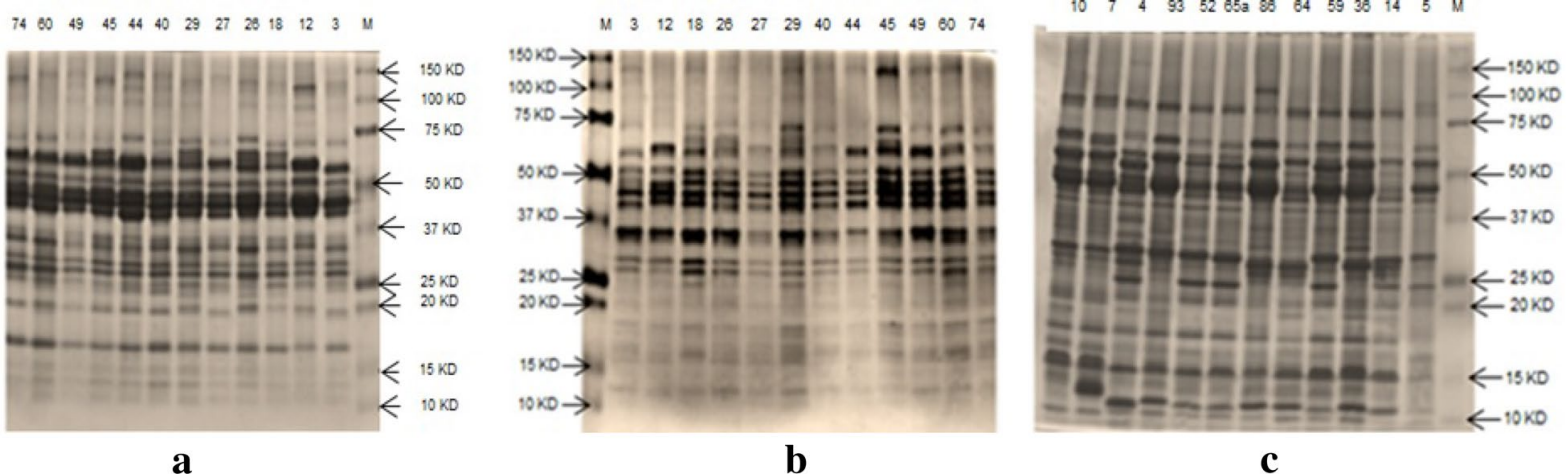
Electrophoretic banding pattern generated by SDS-PAGE of seed storage proteins of some *Vicia* accessions studied. *M* marker, **a** globulins, **b** prolamins, **c** albumins

**Fig. 3:**
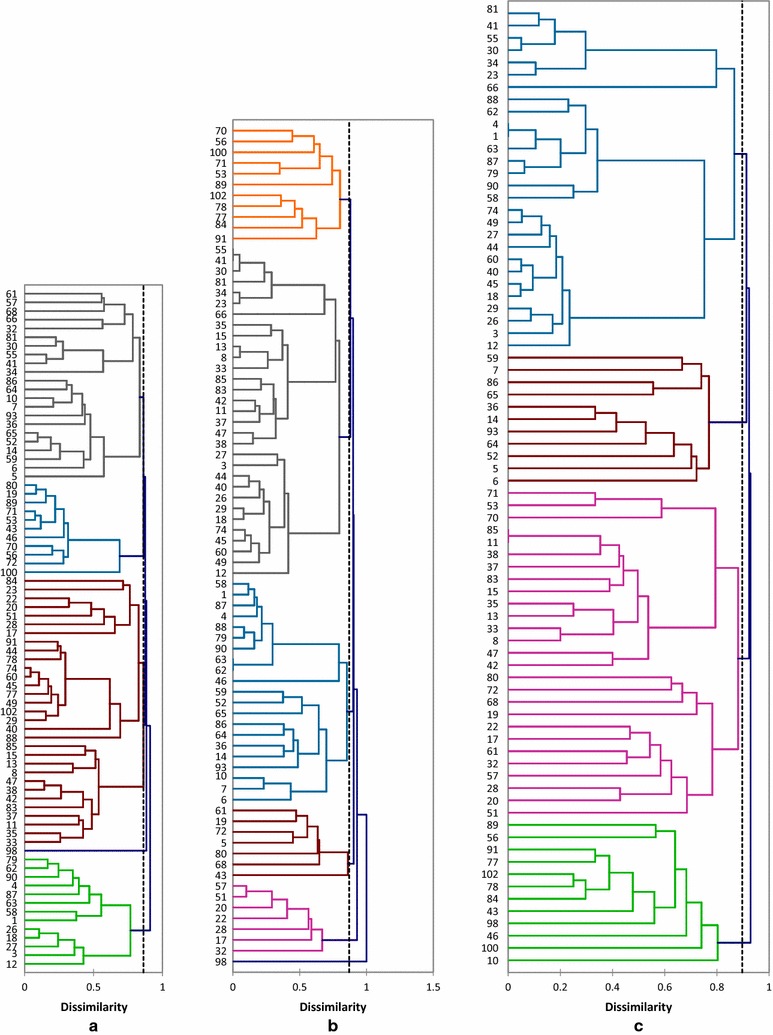
Dendrograms generated using UPGMA cluster analysis and Jaccard’s index based on seed proteins diversity of 78 *Vicia* accessions. **a** Albumins, **b** globulins, **c** prolamins

The three fractions electrophoregrams are represented by some accessions illustrated in Fig. 2. Figure 3 presents dendrograms generated using UPGMA and Jaccard’s index.

#### Albumins patterns

A total of 131 bands were detected with molecular weights ranged from 3.23 to 148.17 kDa. Each profile presents between 11 and 26 bands. All samples had more than one seed protein pattern. Intra-accessional diversity was also investigated by examining populations of the same taxa. The largest number of bands (25) is obtained in *V. monantha* subsp. *calcarata, V. tenuifolia* and *V. sativa* subsp. *obovata*. The lowest number (11) is obtained in *V. lutea* subsp*. vestita*. The band 37.73 kDa is the most common as it appears in 48 profiles followed by the band 9.19 kDa appeared in 43 profiles and the band 24.22 kDa observed in 40 profiles. In parallel, bands 3.23, 9.46, 15.81, 19.80, 24.87, 25.97, 46.86, 63.69, 66.34, 76.29, 99.86, 113.50 and 125.98 kDa are the least common as they are specific for one accession and appear each in 1 profile, followed by bands 8.85, 14.93, 16.72, 42.84, 75.43, 77.31, 82.63 and 88.47 kDa appeared each in two profiles. The cluster analysis indicated the discrimination into five groups at 0.86 Jaccard distance (Fig. 3). The first cluster can be divided into two groups, the first one includes accessions 12, 3, 27, 18, 26. The second one regroups samples 1, 58, 63, 87, 4, 90, 62 and 79. The cluster II is divided into 2 sub-clusters. The first one contains samples 5, 6, 59, 14, 52, 65, 36, 93, 7, 10, 64 and 86. The second one can further be divided into two groups: II2a contains accessions 34, 41, 55, 30 and 81. II2b is composed of samples 32, 66, 68, 57, 61. The cluster III regroups two sub-clusters. The first one contains accessions 33, 35, 11, 37, 83, 42, 38. The second one is further divided into two groups: III2a comprises sample 88 linked to 40, 29, 102, 49, 77, 45, 60, 74, 78, 44 and 91. III2b contains accessions 17, 28, 51, 20, 22, 23 and 84. The cluster IV comprises the sample 100 one side and accessions 72, 56, 70, 46, 43, 53, 71, 89, 19 and 80 another side. Finally, the cluster V contains the sample 98 (J = 0.88). The proximity matrix using Jaccard index shows that the higher distance (J = 1) is observed between the following couples: 17-4, 1-30, 1-32, 1-37, 1-41, 1-81, 4-28, 4-32, 4-84, 6-63, 6-87, 7-87, 63-10, 87-10, 14-63, 14-87, 17-63, 28-63, 30-58, 32-58, 62-32, 79-32, 32-90, 37-90, 58-37, 37-62, 41-58, 41-1, 52-87, 55-1, 55-58, 58-81, 66-58, 58-41, 58-30, 58-32, 59-63, 87-59, 63-93, 63-84, 63-7, 63-23, 63-52, 63-64, 63-65, 64-87, 65-87, 1-66, 79-32, 87-93, 87-65. The lower distance (J = 0.10) is obtained between 18 and 26. A distance of 0.16 is observed between 43 and 19. Between 89 and 19, a distance of J = 0.15 is observed. A distance of 0.17 is obtained between 90 and 62.

#### Globulin patterns

A total of 119 bands were obtained with molecular weights ranged from 2.77 to 131.88 kDa. Each profile presents between 6 and 27 bands. Two accessions (41 and 55) showed a unique protein pattern; the remaining accessions had more than one protein pattern. The largest number of bands (27) is observed for accessions 43 (*V. monantha* subsp. *calcarta)* and 87 (*V. lutea* subsp*. eu*-*lutea).* The lowest number (6) is obtained for samples 17 and 32 belonging to *V. sativa* subsp*. obovata.* The band 35.53 kDa is the most common as it appears in 52 profiles, followed by the band 49.45 kDa observed in 45 profiles and the band 33.44 kDa found in 37 profiles. In another side, bands 5.10, 5.46, 51.74, 67.97, 78.27, 85.47, 95.33, 100.77, 103.66, 104.69, 115.04, 119.96, 130.35 kDa are the less common as they appear each in one profile, followed by bands 16.86, 18.39, 19.11, 28.98, 37.69, 47, 38.87, 68.53, 86.70 and 131.88 kDa found in two profiles. Six major clusters were obtained at the distance of 0.87 (Fig. 3b). The cluster I is further divided into two sub-clusters (I1, I2). I1 includes the accession 46 linked to 62, 63, 90, 79, 88, 4, 87, 1 and 58. I2 can be divided into two groups. The first one (I2a) includes 59, 52, 65, 86, 64, 36, 14 and 93. The second one (I2b) contains samples 6, 7 and 10. The cluster II is divided into two subclusters (II1, II2). II1 comprises two groups. II1a contains accessions 55, 41, 30, 81, 34, 23. II1b is composed of 35, 15, 13, 8, 33, 85, 83, 42, 11, 37, 47, 38. II2 comprises sample 12 linked to 49, 60, 45, 74, 18, 29, 26, 40, 44, 3 and 27. Cluster III contains sample 43 linked to accessions 68, 80, 5, 72, 19 and 61. The cluster IV (J = 0.92) comprises samples 32, 17, 28, 22, 20, 51 and 57. The cluster V (J = 0.87) is divided into two sub-clusters. V1 includes 70, 56, 100, 71, 53 and 89. V2 contains samples 102, 78, 77, 84 and 91. The accession 98, being itself the cluster VI at J = 1. The dissimilarity matrix shows that a distance of 0.00 is observed between couples: 62-63, 55-41. A low distance of 0.11 is observed between samples 1 and 58. Between accessions 1 and 4, a distance of 0.14 is observed. Samples 58 and 4 are distant by J = 0.18. A distance of 0.14 is obtained between 1 and 87. Samples 4 and 88 have a distance of 0.15. The higher distance (J = 1) is observed between 98 and all other accessions. The same distance is observed for the following couples: 5-66, 5-34, 5-22, 5-23, 10-20, 10-22, 72-17, 68-17, 46-17, 43-17, 19-17, 81-19, 84-19, 66-19, 65-19, 55-19, 57-19, 51-19, 41-19, 32-19, 34-19, 23-19, 20-52, 22-80, 22-72, 22-68, 22-62, 22-63, 22-52, 22-46, 22-28, 22-19, 19-30, 32-43, 32-46, 32-68, 32-71, 46-51, 46-57, 51-68, 51-72, 57-72, 93-61, 100-65, 93-80, 98-100 and 98-102.

#### Prolamin patterns

A total of 98 bands were obtained with molecular weights ranged from 11.36 to 137.638 kDa. Each profile presents between 6 and 24 bands. Two accessions (1 and 4) showed a single protein pattern; the remaining accessions had more than one seed protein pattern. The largest number of bands (24) is observed in patterns 58 (*V. lutea* subsp*. vestita)* and 62 (*V. lutea* subsp*. eu*-*lutea).* The lowest number (6) is obtained for samples 19 (*V. sativa* subsp. *angustifolia*), 7 and 51 both belonging to *V. sativa* subsp. *obovata*. The band 17.65 kDa is the most common as it appears in 46 profiles followed by the band 41.49 kDa appeared in 39 profiles and the band 35.53 kDa observed in 29 profiles. Bands 12.44, 51.51, 53, 56.62, 78.57, 83.92, 89.03, 94.53, 104.51, 105.45, 115.08, 118.17 and 137.63 kDa are the less common as they appear in one profile each, followed by bands 29.49, 66.63, 68.33, 81.77, 85.10, 106.71, 109.03 and 127.97 kDa obtained each in two profiles. The UPGMA generated four major clusters at the distance of J = 0.89. The first cluster (I) is divided into two sub-clusters (I1, I2). I1 includes samples 81, 41, 55, 30, 34, 23 and 66. I2 can further be divided into two groups (I2a, I2b). I2a contains accessions 88, 62, 4, 1, 63, 87, 79, 90 and 58. I2b is composed of 74, 49, 27, 44, 60, 40, 45, 18, 29, 26, 3 and 12. The cluster II comprises two sub-clusters (II1, II2). II1 contains accessions 59, 7, 86, 65. II2 includes 36, 14, 93, 64, 52, 5 and 6. The cluster III (J = 0.92) comprises two sub-clusters (III1, III2). III1 is composed of accessions 71, 53 and 70 in the group III1a and accessions 85, 11, 38, 37, 83, 15, 35, 13, 33, 8, 47 and 42 in the group III1b. The sub-cluster III2 includes two groups. III2a contains accessions 80, 72, 68 and 19. III2b is composed of 22, 17, 61, 32, 57, 28, 20 and 51. The cluster IV includes samples 89, 56, 91, 77, 102, 78, 84, 43, 98, 46, 100 and 10. The proximity matrix using Jaccard’s index shows that a distance of J = 0 is observed between samples 1 and 4. The same distance is obtained between accessions 11 and 85. Between accessions 40 and 60, a distance of 0.05 can be observed. A distance of 0.08 is obtained between samples 26 and 29. A low distance of 0.10 can be observed for the couples: 1-63, 4-63, 23-34, 55-81, 60-74. Between samples 3 and 29, 63 and 87, a distance of J = 0.16 is observed. Samples 41 and 81, 63 and 79 are distant by 0.11. A distance of 0.17 is obtained for: 3-26, 26-18, 49-29. Samples 49 and 12 are distant by J = 0.19. A distance of 0.18 is observed between accessions 12 and 26 and accessions 26 and 49. Accessions 26-27, 26-74 and 45-49 are distant by J = 0.14. A distance of 0.15 is observed for accessions 27-49 and 30-81. Couples 29-60 and 29-45 have a distance of 0.13. The higher distance (J = 1) is obtained for a large number of couples as for: 1-84, 1-100, 3-65, 3-59, 3-32, 3-28, 3-20, 4-84, 4-100, 5-100, 5-80, 5-72, 5-68, 5-61, 5-57, 5-51, 5-43, 5-34, 5-32, 5-28, 5-23, 5-19, 5-20, 5-22, 5-17, 6-43, 6-102, 7-102, 7-100, 7-91, 7-84, 7-77, 7-78, 7-66, 7-61, 7-46, 7-51, 7-43, 7-32, 7-33, 7-28, 7-20, 7-22, 7-17, 7-13, 7-8, 8-19, 8-43, 8-46, 8-56, 8-59, 8-65, 8-77, 8-78, 8-84, 8-86, 8-91, 8-98, 8-100, 8-102, 10-61, 10-57, 10-32, 10-28, 10-20, 11-19, 11-43, 11-46, 11-56, 11-59, 11-65, 11-77, 11-78, 11-84, 11-86, 11-91, 11-98, 11-100, 11-102, 12-65, 12-61, 12-59, 12-51, 12-32, 12-28, 12-19, 12-20, 13-19, 13-46, 13-56, 13-59, 13-65, 13-77, 13-98, 13-100, 13-72, 13-68, 13-61, 13-46, 13-51, 13-43, 13-32, 13-28, 13-20, 13-22, 13-17, 15-59, 15-65, 15-86, 17-86, 17-64, 17-65, 17-59, 17-52, 17-36, 18-20, 18-28, 18-32, 18-51, 18-59, 18-61.

### Mantel test

A Mantel test based on Pearson’s correlation was used to highlight correlations between the matrices of albumins (matrix A), globulins (matrix B) and prolamins (matrix C). The *p* value was calculated from the distribution of r(AB) using 10,000 permutations with the value of r(AB.C) = 0.3099. This test showed significant correlation between the three fractions studied since the calculated p-value (<0.0001) is below the significance level of alpha (0.05 = 5%). Concerning the correlation between ecogeography and seed proteins, r values were −0.0012, −0.0039 and 0.0166 respectively for albumins, globulins and prolamins. *p*-values are 0.8233, 0.9319 and 0.3689 respectively for the three fractions. Thus, Mantel test showed no significant correlation between ecogeography and protein patterns since the calculated *p*-values are below the significance level of alpha.

### Cluster analysis based on ecogeographic data

**Fig. 4:**
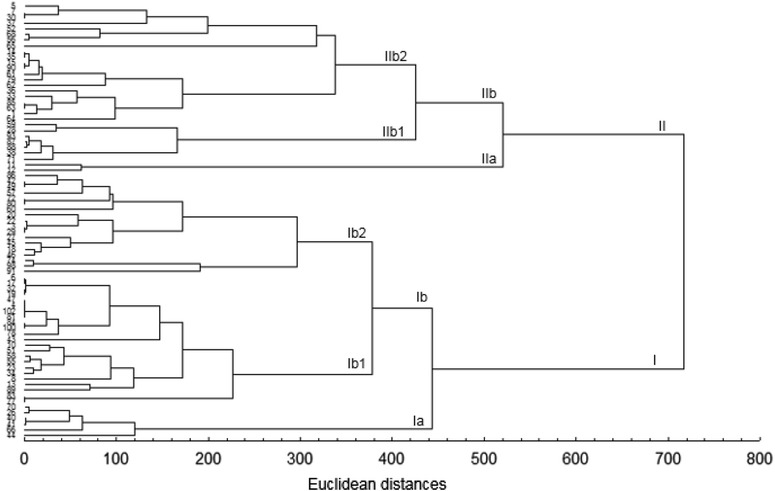
Dendrogram generated using UPGMA cluster analysis and Euclidean distances based on ecogeographic characterization of sites investigated

The dendrogram illustrated in Fig. 4 shows the relationships between these taxa, based on the variation in the five ecogeographic parameters studied. At the Euclidean distance of 716.43, the dendrogram can be divided into two major clusters (I and II). The first one is further divided into two sub-clusters (Ia and Ib). Ia (d = 119.65) comprises samples 44, 66, 41, 40, 26 and 70 belonging to 2 bioclimates (LH–SH). Ib can be divided into Ib1 (d = 226.96) and Ib2 (295.93). Ib1 contains the sample 77 (HA) linked to accessions 83, 89, 13, 8, 34, 23, 55, 58, 51, 10, 43, 78, 100, 84, 81, 102, 4, 47, 19, 32, 17 and 6 from 4 bioclimates (HA–MSA–SH–HSA). Ib2 (d = 295.93) is composed of samples 91, 98, 74 belonging to 2 bioclimates (SH–HSA) linked to 46, 18, 45, 27, 29, 3 (MSA) then 22, 20, 60, 80, 72, 57, 49, 42 and 86. (MSA–LSA–HSA–SH). The second cluster comprises subclusters IIa which contains samples 12 (LSA) and 11 (HA) and IIb. The latter comprises groups IIb1 (d = 165.56) composed of accessions 71, 38, 88, 87, 93, 28 and 59 belonging to 1 bioclimate (LH) and IIb2 which can further be divided into two groups: At a distance of d = 172.22, the first group contains accessions 64, 1, 63, 85, 33, 36, 62, 79, 61, 90, 15, 35 and 14 collected from two bioclimates (LH–SH). The second group (d = 317.53) comprises sample 65 (LH) linked to accessions 56, 68, 52, 37, 30, 7 and 5 (SH–MSA–SA). The higher distance (d = 1286) is observed between 91 (*V. monantha* subsp. *cinerea,* SH) and 93 (*V. sativa* subsp. *consobrina,* LH). A distance of d = 1284 is obtained between 87 (*V. lutea* subsp*. eu*-*lutea,* LH) and 91. The distance of d = 0 is obtained between the following couples: 35-14, 6-17, 32-19, 80-72, 15-90, 42-49, 47-4, 47-102, 47-81, 85-63, 4-102, 77-83, 102-81, 3-29, 30-7, 100-84 and 4-81. Low distances of d = 1 are observed between 6 and 19, 17 and 19, 41 and 40.

## Discussion

The discrimination in the genus *Vicia* into subgenera, sections and subsections was undertaken by several studies, based on morphological and cytological analyses (Hanelt and Mettin 1989; Kupicha 1976; Leht 2009). In the present work, seed storage proteins and ecogeographic parameters were used.

### Seed proteins variability

The differences among accessions were observed and all eleven taxa can be recognized by their protein profiles. Samples within each taxon showed a different number of bands with different molecular weights. Thus, intraspecific heterogeneity is obtained. A positive correlation was exhibited between seed globulin, seed albumin and seed prolamin contents (highly significant). Our results partially confirmed classification of *Vicia* by Kupicha (1976), Hanelt and Mettin (1989) and Leht (2009) at subgeneric and sectional levels. According to Osborne (1924), proteins are classified into albumins, globulins, prolamins and glutelins based on their solubility which is a convenient method to initiate the discrimination of the seed storage proteins from a species that has not been studied in detail (Ribeiro et al. 2004). The differences in the three fractions profiles of individual seeds was expected since Mudzana et al. (1995) and Goodrich et al. (1985) found that there was variability in the total seed storage protein profiles of individual seeds within a subspecies. This was probably due the cross fertilization nature of the genus.

#### Albumins patterns

Albumins cluster analysis revealed five major clusters, differing only in the relative position of some accessions in subgroups. Populations of *V. monantha* subsp. *calcarata* belonging to section Cracca of the subgenus Vicilla (sensu Kupicha) are linked to samples of *V. lutea* (Sect. Hypechusa) which indicates a close relationship between the two subspecies of *V. lutea* when it is difficult to determine distinct groups which could be individually identified as *eu*-*lutea* or as *vestita*. There are bands which are specific of some accessions and can be used as markers to discriminate samples at interspecific level. Discrimination at intraspecific level is also obtained by albumins patterns. The use of albumins proved to be helpful in revealing interspecific variability and intraspecific diversity in the studied taxa. Some bands are specific constant markers for each taxon and can be discriminated bay their electrophoregrams. Other bands are common of several taxa. Albumins present the highest bands number which indicates major role of albumin heterogeneity in discriminating the *Vicia* samples (Mustafa 2007).

#### Globulins patterns

Cluster analysis of globulins patterns revealed six basic groups. A low distance can be observed between samples of *V. lutea* subsp. *vestita* or by samples of *V. lutea* subsp. *eu*-*utea* and *V. lutea* subsp. *vestita. V. narbonensis* (sect. Narbonensis) and *V. lutea* subsp. *vestita* (sect. Hypechusa) present close globulin profiles. These results concord with those of Jaaska (1997), Jaaska and Leht (2007) and Shiran and Raina (2014), which showed the species of sections Hypechusa as sister to the clade of section Narbonensis. High distances are observed between *V. narbonensis* (sect. Narbonensis) and *V. sativa* (sect. Vicia) and between species of *V. sativa* or between *V. lutea* subsp. *eu*-*lutea* (sect. Hypechusa) and *V. sativa.* Globulins are the major storage proteins present in seeds of legumes (Freitas et al. 2000) and differences both at intraspecific and interspecific levels can be obtained by globulins patterns. A good example in this case is the one of sample 43 (*V. monantha* subsp. *calcarata*) characterized by 3 specific bands (51.74, 78.27 and 115.04 kDa). The unique population of *V. leucantha* also has a specific band (119.96 kDa). Samples of *V. monantha* subsp. *calcarata* are a good example for intraspecific heterogeneity as shown by accessions 102 and 43, characterized by one specific band each.

#### Prolamins patterns

Cluster analysis of prolamins patterns revealed four basic groups. Few studies have been reported concerning the utilization of prolamins patterns to discriminate *Vicia* taxa in comparison with albumins and globulins. The classification obtained using the UPGMA showed that samples belonging to the same taxon close together in the clusters. A common profile was observed for samples 1 and 4 belonging to *V. lutea* subsp. *vestita*, but this taxon also showed other patterns. It is may be due to the fact that native wild populations are composed of a mixture of genotypes which provide survival advantages in varied environmental conditions. Outcrossing could also be an explanation of diversity in the accessions studied, as indicated for several types of vetch (Hanelt and Mettin 1989; Mirali et al. 2007). The sample 10 (*V. sativa* subsp. *obovata*) showed differences in its electrophoregram compared to the other taxon members, and might be considered an ‘‘off type’’ as proposed by De la Rosa and Gonzalez (2010). Prolamins patterns are a good discriminatory marker in *Vicia* taxa at both intraspecific and interspecific levels especially for *V. sativa* samples as the subspecies belonging to this species can be characterized by specific bands as in the case of samples 6, 10, 57, 82 and 95 of *V. sativa* subsp. *obovata* or samples 48, 77 and 42 belonging to *V. sativa* subsp*. cordata*. *V. leucantha*, *V. tenuifolia* and *V. narbonensis* are also characterized by specific bands which could be considered as markers at interspecific level.

### Interspecific and intraspecific variation

#### V. sativa s.l. (Sect. Vicia)

*Vicia sativa* is the most polymorphic species of the genus *Vicia* and the debate about its taxonomic classification is extensive. In the present work, the all studied taxa of *V. sativa* s.l (section *Vicia*) are found in the same group on the basis of albumins homology and can be found in three clusters which indicates a close relationship between subspecies of *V. sativa* when it is difficult to observe separate groups which could be identified as *obovata, consobrina, cordata* or *angustifolia*. In our previous paper using plant morphology (Bechkri and Khelifi 2016), our results have demonstrated that in the *V. sativa* using morphological traits alone do not provide a stable grouping. A close relationship between samples can be seen. The picture generated between the phylogenetic trees may be due to the possible phylogenetic instability of these taxa as indicated by Leht (2009).

#### V. narbonensis (Sect. Narbonensis) and V. lutea (Sect. Hypechusa)

On the basis of albumins patterns, all populations of *V. narbonensis* belong to the same subcluster except for the accession 23 which is linked to accessions of *V. sativa*. Globulins patterns linked all accessions of *V. narbonensis* together. Accessions of *V. narbonensis* clustered together using prolamins profiles. Albumins, globulins and prolamins patterns joined all samples of *V. lutea* in the same subcluster with no discrimination between subsp. *eu*-*lutea* and subsp. *vetsita.* These observations show that there is an overlap between accessions of these two subspecies. The utilization of seed storage proteins shows a close relationship between the taxa when it is difficult to distinguish groups which could be identified as *eu*-*lutea* or as *vestita*. Albumins and globulins profiles not link accessions of *V. lutea* and *V. narbonensis.* Prolamins patterns of the present study concord with those of Jaaska (1997) and Jaaska and Leht (2007) and Shiran and Raina (2014) which showed the species of sections Hypechusa as sister to the clade of section Narbonensis. Our data revealed that subgenus *Vicia* is a well-separated subgenus and agreed with the results based on morphology reported by Diklic (1972) and with results on phylogenetic relationships (Potokina et al. 1999; Leht 2009). Seed albumins, globulins and prolamins patterns showed *V. lutea* samples to form an homogenous group. The same findings were reported by Przybylska and Zimniak-Przybylska (1997).

#### V. monantha, V. tenuifolia and V. leucantha (Section Cracca)

Samples of *V. monantha* clustered together in two different groups on the basis of albumins patterns with no distinction between the two subspecies *calcarata* and *cinerea.* An exception is observed for two samples (43 and 46) which clustered with accessions of *V. sativa*, *V. tenuifolia* and *V. leucantha*. Globulins patterns joined the majority of samples of *V. monantha* subsp. *calcarata* all together with an exception for the sample 98 which forms a separate cluster. Profiles of *V. monantha* subsp. *cinerea* do not have provided clear groupings. Prolamins patterns gave stable groups where samples of subsp. *monantha* are linked together. Six samples clustered together in another subcluster which contains samples of subsp. *cinerea.* Samples of these species clustered together on the basis of albumins, globulins and prolamins patterns. They are also linked to samples of *V. monantha.* Thus, species of section Cracca sensu Kupicha belong to one group. Populations of *V. tenuifolia*, *V. leucantha* and *V. monantha* clustered together showing also an overlap between these taxa. These species are classified by Kupicha (1976) in the sub-genus *Vicilla*, section Cracca. Thus, the three species attributed to the section Cracca are joined in a separate group in the present work. *V. tenuifolia* and *V. leucantha*, are grouped in one subcluster of closely related taxa that provided strong homologous variation with shared characters. As a consequence, the treatment of *V. tenuifolia*, *V. monantha* and *V. leucantha* in the section *Cracca* is supported. In the present analysis, *V. leucantha*, the species transferred by Ball (1968) to his section Ervum, is in the same clade with the remaining Cracca species. In spite of this, our analysis of seed proteins supports Kupicha’s placement of *V. leucantha* in section Cracca as was also done by Davis and Plitmann (1970).

### Ecogeographic characterization

As the first step towards more efficient conservation is to undertake an ecogeographic study (Maxted et al. 1996), the aim of the present work was to collect ecogeographic informations from investigated stations of *Vicia* L. Analysis of the passport data will elucidate each taxon’s geographic and ecological location. The distribution maps will be used in the planning of future collecting missions. The wide geographic ranges may explain the high degree of protein seed storage variation among accessions and should be considered in conservation programs of this *Vicia* taxa (El Bakatoushi and Ashour 2009). The obtained intraspecific diversity within the taxa reflects a wide geographical and ecological distribution of this species as reported by Ehrman and Maxted (1990) and Maxted (1995). Studying the species from different geographic regions and altitudes, indicates that the species may be still evolving in different pathways as reported by Ashour et al. (2005). According to Hannelt and Mettin (1989), *Vicia* taxa do not tolerate extreme environmental conditions. Whereas, Francis et al. (2000) report that *V. sativa* has good adaption to adverse environmental conditions. Cluster analysis shows that samples having differences in electropherograms and belonging to different taxa can belong to an identical bioclimate and altitudes as in the case of accessions 19 and 32 which were collected from the same locality. In parallel, there are samples with high protein homology level which are collected from stations belonging to the same bioclimate. Accessions 72 and 80 are a good example in this case. The dendrogram obtained with ecogeographic parameters did not indicate clear discrimination among accessions based on their geographical locations. The Mantel test between proteins patterns and ecogeography indicated that the correlation between proteins profiles resemblance and geographical origin is less significant. The same findings were reported by Chung et al. (2013), Potokina et al. (2003), Mirali et al. (2007) and De la Rosa and Gonzalez (2010). Considering all stations of the current paper, the studied samples of *Vicia* L. occur from 1 to 1222 m. Stations belong to seven different bioclimates (SH, LH, HSA, MSA, LSA, HA) and are characterized by cool, wild, warm or temperate winters. Considering all stations of the current work, *V. sativa* L. occurs from sea level to 880 m which is consistent with the findings of Maxted (1995). Samples of *V. narbonensis* were collected from sites receiving between 382.95 and 697.18 mm of precipitations and belonging to bioclimates characterized by cool or mild winter. Bennett and Maxted (1997) reported that the *V. narbonensis* occur over a wide range of altitudes, from sea level to 3200 m when Abd El Moneim (1992) reported that *V. narbonensis* adapts in areas receiving 250–300 mm annual precipitations and are characterized by low winter temperatures. Accessions of *V. lutea* were collected from sites belonging to four bioclimates (LH, HSA, MSA, SH) and altitudes ranged between 11 and 604 m with a minimum temperatures ranged from 2.55 to 8.64 °C. Accessions 74, 77, 91 and 98 belonging to *V. monanatha* occur until 1222 m and at these altitudes, they require some frost tolerance as the temperatures can drop to 0.63 °C as in the case of the accession 98. *V. leucantha* was collected from a site characterized by HSA bioclimate with a minimum temperature of 2.62 °C (cool winter) and an altitude of 586 m. The two samples of *V. tenuifolia* occurred in SH and MSA bioclimates with mild or cool winters and at altitudes of 276 and 543 m. These patterns are not necessarily a real picture of the preferred altitude of these two taxa. A larger number of accessions from each geographical location should be tested to confirm patterns.

## Conclusion

The electrophoregrams obtained can be exploited as passport data for the genetic diversity of the studied taxa. Seed protein electrophoresis is a valid tool for taxa discrimination. The variability observed indicates that improvement by simple selection for these traits is possible. No significant correlation is obtained between seed proteins and ecogeography. The use of more samples from different origins is necessary to include most of the genetic determinants of these traits.
